# Neoadjuvant irradiation of extremity soft tissue sarcoma with ions (Extrem-ion): study protocol for a randomized phase II pilot trial

**DOI:** 10.1186/s12885-022-09560-x

**Published:** 2022-05-12

**Authors:** D. Brügemann, B. Lehner, M. Kieser, J. Krisam, A. Hommertgen, C. Jaekel, S. B. Harrabi, K. Herfarth, G. Mechtesheimer, O. Sedlaczek, G. Egerer, A. Geisbüsch, M. Uhl, J. Debus, K. Seidensaal

**Affiliations:** 1 Department of Radiation Oncology, Heidelberg University Hospital, Im Neuenheimer Feld 400, 69120 Heidelberg, Germany; 2 Heidelberg Institute of Radiation Oncology (HIRO), Heidelberg, Germany; 3 National Center for Tumor diseases (NCT), Heidelberg, Germany; 4 Department of Radiation Oncology, Heidelberg Ion-Beam Therapy Center (HIT), Heidelberg University Hospital, Heidelberg, Germany; 5 Center for Orthopedics, Trauma Surgery and Paraplegiology, University of Heidelberg, Heidelberg, Germany; 6 Institute for Medical Biometry, University of Heidelberg, Heidelberg, Germany; 7 Clinical Cooperation Unit Radiation Oncology, German Cancer Research Center (DKFZ), Heidelberg, Germany; 8 Institute of Pathology, University of Heidelberg, Heidelberg, Germany; 9 Department of Radiology, University of Heidelberg, Heidelberg, Germany; 10 Department of Radiology, German Cancer Research Center (DKFZ), Heidelberg, Germany; 11 Department of Hematology, Oncology and Rheumatology, Heidelberg University, Heidelberg, Germany; 12 Department of Radiation Oncology, Klinikum Ludwigshafen, Ludwigshafen, Germany; 13 German Cancer Consortium (DKTK), Heidelberg, Germany

**Keywords:** Extremity soft tissue sarcoma, Carbon ion therapy, Proton therapy, Irradiation, Randomized trial, Hypofractionation, Heavy ion therapy

## Abstract

**Background:**

The standard of care treatment for soft tissue sarcoma of the extremities is a wide resection in combination with pre- or postoperative radiotherapy with high local control rates, sparing patients the necessity of amputation without compromising on overall survival rates.

The currently preferred timing of radiotherapy is under debate. Albeit having higher rates of acute wound complications, late side effects like fibrosis, joint stiffness or edema are less frequent in preoperative compared to postoperative radiotherapy. This can be explained in smaller treatment volumes and a lower dose in the preoperative setting. Particles allow better sparing of surrounding tissues at risk, and carbon ions additionally offer biologic advantages and are preferred in less radiosensitive tumors. Hypofractionation allows for a significantly shorter treatment duration.

**Methods:**

Extrem-ion is a prospective, randomized, monocentric phase II trial. Patients with resectable or marginally resectable, histologically confirmed soft tissue sarcoma of the extremities will be randomized between neoadjuvant proton or neoadjuvant carbon ion radiotherapy in active scanning beam application technique (39 Gy [relative biological effectiveness, RBE] in 13 fractions [5–6 fractions per week] in each arm). The primary objective is the proportion of therapies without wound healing disorder the first 120 days after surgery or discontinuation of treatment for any reason related to the treatment. The secondary endpoints of the study consist of local control, local progression-free survival, disease-free survival, overall survival, and quality of life.

**Discussion:**

The aim of this study is to confirm that hypofractionated, preoperative radiotherapy is safe and feasible. The potential for reduced toxicity by the utilization of particle therapy is the rational of this trial. A subsequent randomized phase III trial will compare the hypofractionated proton and carbon ion irradiation in regards to local control.

**Trial registration:**

ClinicalTrials.gov Identifier: NCT04946357; Retrospectively registered June 30, 2021.

## Background

Soft tissue sarcomas (STS) collectively account for approximately 1% of all adult malignancies [1]. There are more than 50 subtypes, while pleomorphic sarcoma, liposarcoma, leiomyosarcoma, synovial sarcoma, and malignant peripheral nerve sheath tumor account for 75% of STS [2]. With 43% the most common localization for STS are the extremities. Limb sparing wide resection in combination with radiotherapy (RT) is the current standard of care for high grade soft tissue sarcomas of the extremities [3]. Especially patients with G2/G3 sarcomas profit from the combination of RT and surgery [4]. Well-differentiated sarcomas (G1) receive no subsequent treatment after total resection (R0). Compared to amputation limb sparing resection with adjuvant RT shows similar overall survival (OS) und disease free survival (DFS) rates with a high local tumor control (LC) according to prospective studies [4–6] and a recent meta-analysis [7]. The status of postoperative resection margins is the most important prognostic factor contributing to LC with local recurrence (LR) being up to 3.76 times more likely in patients with positive compared to negative resection margins [8]. The sequence of surgery and RT is widely discussed by the radiation oncologists and surgeons. The main advantages of neoadjuvant (preoperative) radiotherapy are the smaller treatment target volumes and reduced prescribed radiation doses of 50 Gy vs. 66 Gy in 2 Gy single doses in the postoperative setting. Both preoperative and postoperative RT show a similar improvement in LC in prospective studies [4, 9–12]. In a recent retrospective study preoperative RT was predictive for R0 resection [13]. However, a greater incidence of acute wound complications was associated with preoperative compared to postoperative RT (35% vs.17%). Late-treatment–related side effects were more frequent in patients receiving postoperative RT, which could be explained by the higher RT dose and the larger treatment volume [10, 14]. The special physical and biological properties of protons and carbon ions lead to a superior dose distribution [15, 16]. This allows a better sparing of the surrounding tissue including the skin, muscles, joints, bones and subcutaneous fatty tissue, potentially reducing acute postoperative wound complications or late treatment related toxicities like fibrosis, joint stiffness, bone fracture or edema. Carbon ions are furthermore superior to protons based on their enhanced biological effectivity, which qualifies those for the treatment of tumors of low radiosensitivity as sarcomas. The standard neoadjuvant normofractionated radiotherapy consists of 25 fractions with 2-Gy single dose over 5 weeks; the special properties of particles allow hypofractionation resulting in a reduced treatment duration of less than 3 weeks.

This single-institution, prospective, randomized, open-label phase 2 trial evaluates the safety and feasibility of a hypofractionated, neoadjuvant proton or carbon ion radiotherapy based on the rate of wound healing disorders.

## Methods

### Primary objective

The primary objective of this trial is the evaluation of safety and feasibility of neoadjuvant hypofractionated irradiation in patients with extremity sarcoma using ions (protons or carbon ions) in raster scan technique. The primary endpoint is defined as the rate of wound healing disorders from beginning of radiotherapy to maximum 120 days after the planned tumor resection or discontinuation for any reason related to the treatment.

### Secondary objectives

Secondary objectives include the assessment of local control (LC), local progression-free survival (LPFS) and disease-free survival (DFS). Overall survival (OS) will be calculated from the start of treatment until death or censoring. Quality of life (QoL) will be assessed using the EORTC-QLQ30 questionnaire.

### Study design

The study is a parallel-group, prospective, randomized clinical trial with an adaptive two-stage phase II trial design (including a sample size recalculation at the interim analysis) for patients with extremity soft tissue sarcoma, randomized to one of the two treatment arms (arm A: proton therapy, arm B: carbon ion therapy). The total dose of 39 Gy (RBE) in 13 fractions will be prescribed in both arms. The accrual period of this trial will take approximately 2 years with a follow-up time of 12 months for each patient. Patients meeting the eligibility criteria and willing to participate will give written informed consent and will be enrolled at Heidelberg University Hospital.

### Inclusion criteria

i) Histologically confirmed soft-tissue sarcoma of the extremities with an indication for perioperative radiation treatment
ii) Resectable or marginally resectable
iii) Karnofsky index of ≥70%
iv) Age ≥ 18 years
v) Carried out patient education and written consent
vi) Patient is capable to give informed consent

### Exclusion criteria

i) Stage IV (distant metastases)
ii) Lymph node metastasis
iii) Metal implants that influence treatment planning with ions
iv) Previous radiotherapy in the treatment area
v) Desmoid tumors
vi) Simultaneous participation in another clinical trial that could influence the results of the study.
vii) Active medical implants for which no ion beam irradiation permit exists at the time of treatment (e.g., cardiac pacemaker, defibrillator)

### Treatment planning and target volume delineation

Examinations for treatment planning consist of a CT scan (3-mm slice thickness) in treatment position and an MRI for 3D image correlation. The delineation of the extremity soft tissue sarcoma requires a T1-weighted post-gadolinium sequence.

Before performing the planning CT the prospective location of incision for the surgery is marked by the surgeon and subsequently in the contrast enhanced CT represented by a steel wire in order to spare this region in the treatment planning if possible.

**Fig. 1:**
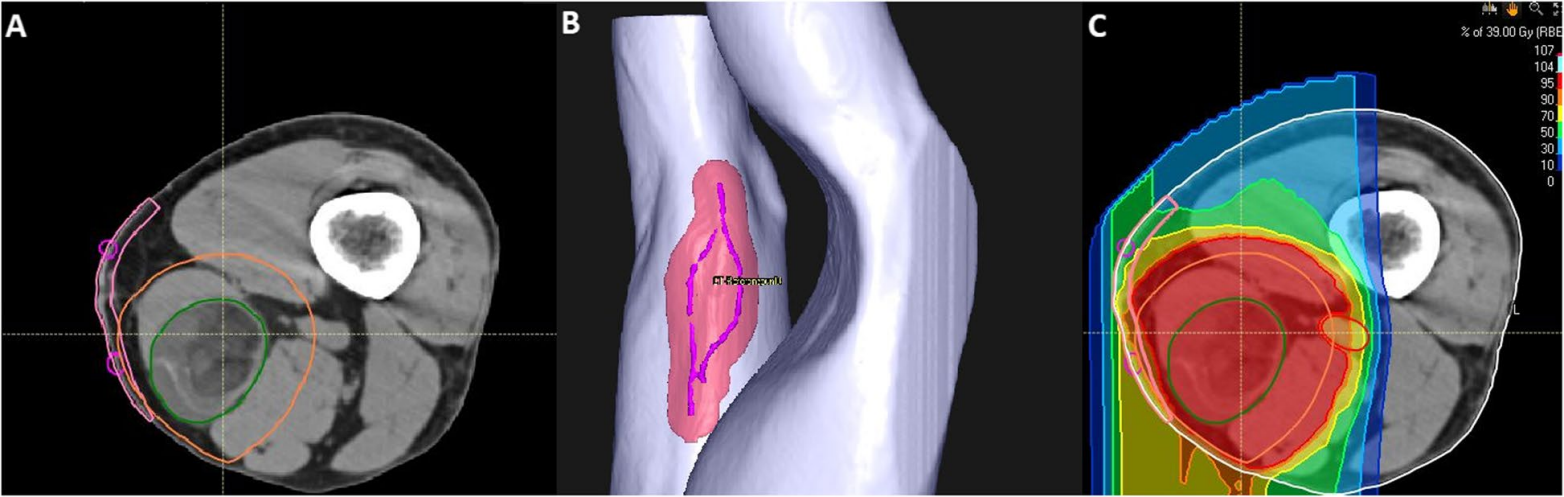
Target volume delineation and treatment planning: **A** The gross target volume (GTV) is depicted in green and encompassed by the clinical target volume in orange (CTV) with a margin of 3-4 cm in the longitudinal and 1.5-2 cm in the lateral direction. **B** The metallic wire marking the planned surgical incision is delineated and encompassed by a 2 cm margin for skin sparing if possible. **C** Dose distribution using opposing beams, the area of beam entrance was chosen outside of the region destined for surgical incision, treatment was performed with carbon ions

Gross tumor volume (GTV) consists of the gross tumor based on CT and MRI imaging. The clinical target volume (CTV) covers the surrounding areas at risk for containing microscopic disease. The CTV includes the GTV with a lateral margin of 1-2 cm and a longitudinal margin of 3-4 cm. CTV extension is limited to the anatomical compartments and includes the tumor surrounding edema. The planning target volume (PTV) includes the CTV with an additional margin of 7 mm in beam direction and 5 mm in all other directions (Fig. 1).

### Proton/carbon ion therapy

Treatment will be administered on an outpatient basis at the Heidelberg Ion-Beam Therapy Center (HIT) [17, 18]. Treatment planning is realized using a treatment planning system (RayStation) that enables conventional and biological optimization. Proton and carbon ion treatment is performed as active beam application (raster scanning method).

### Dose prescription and constraints of critical organs at risk

The prescribed dose to the PTV is 39 Gy (RBE) in 13 fractions (5–6 fractions per week). It is estimated that the α/β value of soft tissue sarcoma is < 10; a consistent value cannot be found in literature. The equivalent photon dose in 2 Gy fractions (EQD2) is in the range of 48.8–42.2 Gy for a α/β = 2–10. There are no risk organs with specific dose constraints to be considered in the extremities.

### Toxicity, safety, and quality of life

Adverse events and toxicity will be reported according to International Common Terminology Criteria for Adverse Events (CTCAE) version 5.0 system. The results of clinical examinations as well as imaging studies (MRI or CT) will be included. Each patient will pass a baseline clinical examination after enrollment. Patients are monitored continuously during treatment, acute adverse events will be documented weekly as well as at the end of treatment. MRI of the according extremity will be planned 3 weeks and the resection 4–6 weeks after the end of radiotherapy. The first clinical follow-up presentation is 6 weeks after resection. Hereinafter all follow-ups include imaging studies and are performed 3, 6, 9, and 12 months after the tumor resection (Table 1). The criteria for feasibility and safety are fulfilled, if the rate of wound healing complications within 120 days after surgery is below 15% and if the therapy was not canceled, unless the reason for canceling is clearly not treatment related.

**Table 1:** Time flow of procedures and examinations

	Study inclusion	Prior to RT	During RT	At the end of RT	3 wk. after RT	OP: 4-6 wk. after RT	6 wk. after RT	3 mo. after OP	6 mo. after OP	9 mo. after OP	12 mo. after OP
Inclusion and exclusion criteria	x										
Informed consent	x										
Medical history	x		x	x	x		x	x	x	x	x
Evaluation of symptoms and toxicity	x		x	x	x		x	x	x	x	x
Radiotherapy			x								
Resection						x					
MRI extremities		x			x			x	x	x	x
CT Thorax		x						x	x	x	x
Quality of life (QLQ-C30)		x			x			x			x

Quality of life (QoL) will be assessed by EORTC QLQ-C30 questionnaires. Repetitive completion of the forms is scheduled before treatment, 3 weeks after the end of radiotherapy, 6 months after surgery, and at the end of the study 12 months after surgery. Statistical analysis will be performed after complete acquisition of data. Changes in QoL before and after radiotherapy and before and after surgery will be determined. The two arms of the study will be compared after the end of radiation and at the end of the whole study.

### Secondary endpoints

The effectiveness of treatment is examined by regular MRI of the according extremity and thoracic CT follow-ups. Local control rate depicts the freedom from local progression. Local progression-free survival is defined as the survival probability from the beginning of radiotherapy until local progression. Disease-free survival is defined as the time from the beginning of radiotherapy until local or distant progression. The overall survival is defined as the time from the beginning of radiotherapy until the date of death due to any reason, until the end of the observation period or until the date of the last presentation in case the patient is lost to follow-up (censoring).

### Statistical methods

This study is performed to deliver basic data for the neoadjuvant hypofractionated carbon ion or proton radiation treatment of patients with extremity soft tissue sarcoma by providing safety and toxicity data for the here presented treatment regimen. The framework of the safety and feasibility considerations is the current standard neoadjuvant normofractionated photon radiotherapy; it is considered as the historical cohort which served for the planning of this pilot study. The randomization will be performed as block randomization at a ratio of 1:1 aiming at equal group sizes by using a web-based randomization tool (randomizer.at).

Due to the pilot character of the trial, all analyses are performed descriptively and the results are to be interpreted accordingly. The primary endpoint is assessed separately in each of the two treatment groups by applying an optimal adaptive two-stage phase II-design with one interim analysis according to Kunzmann and Kieser [19]. This design is an adaptive variant of Simon’s design [20], where the sample size of stage two is chosen optimally based on the results of the interim analysis. It offers the possibility to prematurely terminate the study due to futility or early success at the timepoint of the interim analysis or allows data-driven recalculation of sample size for the second part of the study while adhering to the specified Type I and Type II error rates. The randomization will be performed as block randomization at a ratio of 1:1. After the interim analysis, dependent on the results, different sample sizes in both treatment arms may be required and the randomization ratio would then be adjusted accordingly. Under the null hypothesis, we would expect in both groups wound complications or dicontinuations to occur in ≥35% of the treated patients within 120 days after surgery, so the expected probability of patients with no wound complications and no discontinuation would be π ≤ 65% for both protons as well as carbon ions. Under the alternative hypothesis, we would expect less than 15% of the patients to have wound complications or discontinue therapy within 120 days after surgery with π ≥ 85% being the rate of patients without wound complications or discontinuation within 120 days after surgery.

Given these probabilities of success for the alternative and null hypothesis for the protons and carbon ion group, a statistical power of 1-β = 0.80 and a one-sided Type I error rate α = 0.05 for each of the two comparisons, the expected sample size under the null hypothesis is 25.59 and under the alternative hypothesis it amounts to 29.97; the maximum sample size in this design is 52. The interim analysis is performed when the data of 21 patients are available and therefore this is the minimum sample size. The recalculated sample sizes for stage 2 in both treatment arms depend on the patient number with a treatment success (no wound complications and no discontinuation). These values as well as the boundaries for early stopping and the decision boundaries after stage two are calculated according to Kunzmann and Kieser [19] with program code supplied by the authors. The estimation of success rates in both study arms is performed by calculation of point estimators [21] and calculation of two-sided 95%-Clopper-Pearson-type confidence intervals that take the adaptive two-stage design into account [22].

Analysis of the secondary time-to-event endpoints LPFS, DFS, and OS will be performed by the Kaplan–Meier method, patients without event will be censored. The treatment groups are compared using the logrank test. Quality of life will be measured by the EORTC-QLQ30 questionnaire and assessed by presentation of mean, standard deviation, median, minimum, and maximum as well as application of a two-sample t test. For the secondary endpoint local control, absolute and relative frequencies are calculated for each treatment group together with two-sided 95% Agresti–Coull confidence intervals, and an unconditional fourfold table test will be applied for treatment group comparison. Adverse and serious adverse events will be listed, and absolute and relative frequencies will be calculated.

The primary analysis for the primary and secondary endpoints is based on the intention-to-treat set including all study patients fulfilling the inclusion/exclusion criteria and having been treated for at least 1 week. Additionally, a per-protocol analysis is performed as sensitivity analysis which is based on all patients who have received the treatment as planned and for whom the relevant data is available. Safety endpoints are evaluated for all study patients for which the treatment has started.

Statistical analysis will be performed by the statistical software SAS v9.4 (SAS Institute, Cary, NC).

### Data safety monitoring board

A Data and Safety Monitoring Board (DSMB) is composed of independent experts. The DSMB will monitor the recruitment, the reported (serious) adverse events as well as the quality of data. The goal is to ensure that the study is executed according to current standards of good clinical praxis with focus on the safety interest of the patients. The DSMB will give the principal investigator (PI) recommendations regarding trial modification, continuation, or premature termination.

### Regular study end

The estimated period of patient accrual is 2 years. The regular end of treatment for the individual patient is 2 to 3 weeks after beginning of radiation therapy (13 fractions, 5–6 fractions per week). The regular study end for each patient is after 12 months after surgery. Patients will be then monitored by regular follow-ups according the current guidelines.

### Premature study termination

Reasons for premature termination of the entire study are:

i. Unacceptable risks or toxicities (assessment by DSMB)
ii. One toxicity of grade 5 or two consecutive grade 4 toxicities or five consecutive grade 3 toxicities unequivocally associated with the study therapy. The DSMB assesses and concludes whether a toxicity equal or higher than grade 3 is associated with the study treatment.
iii. New scientific findings incompatible with the study treatment.

### Collection and management of trial-related data

All data are collected pseudonymously and allocated to individual patient numbers. The study data are collected in form of case report forms. All important trial documents will be archived for at least 10 years according to the German GCP-Regulation. The documentation of written informed consent, the patients’ consent for trial and the documentation of irradiation will be archived for 30 years according to the German Radiation Protection Regulation (StrlSchV). The Study Center at the Department of Radiation Oncology will be responsible for storing all relevant data. For scientific evaluation of the study results, the disease-associated data will be saved pseudonymously. Access to the data will be provided to the patient on demand. The principal investigator can grand access to original files, additionally access can be commissioned by the state authorities. If a patient withdraws informed consent and does not agree with further storage and analysis, the so far acquired data material will be destroyed.

The trial is conducted in accordance to their current versions of the Declaration of Helsinki (2008 version of the Declaration of Helsinki, adopted at the 59th WMA General Assembly, Seoul, October 2008) and the guidelines of Good Clinical Practice (ICH-GCP: International Conference on Harmonization - Good Clinical Practice; May 1, 1996).

For comparison please also refer to the trial protocol of the Retro-Ion trial which investigates this radiotherapy regimen of preoperative hypofractionated particle radiotherapy for retroperitoneal sarcoma [23].

## Discussion

The standard of care treatment consisting of limb sparing wide resection and pre- or postoperative photon radiotherapy has achieved satisfactory LC-rates sparing patients the need of amputation without compromising the OS rate [3, 4, 12]. However, the sequence of treatment is still subject of debates. Preoperative radiotherapy is preferred in many guidelines as the National Comprehensive Cancer Network Guidelines (NCCN) over postoperative radiotherapy in order to reduce late toxicities as fibrosis (48% vs. 32%; *p* = 0.07), edema (23.2% vs. 15.5%) as well as joint stiffness (23.2% vs. 17.8%) albeit having higher acute wound complication rates (35% vs.17%) [14]. These late toxicities are often permanent and function limiting. Nonetheless many institutions perform postoperative radiotherapy as acute wound complications are often non-trivial and can require the need for tissue transfer as well as bear the risks for chronic morbidities and the need for subsequent secondary operations. The Canadian Sarcoma Group performed a phase II study for patients with sarcoma of the lower extremity treated with preoperative image-guided intensity modulated radiotherapy (IG-IMRT) and tried to reduce the risk of wound healing disorders by modern conformal photon techniques. Although they could not reach significance, the risk for wound healing disorders was with 30.5% lower compared to the same subgroup with 40.3% for the above-mentioned phase III trial [24]. As the problem remains even with the modern IMRT techniques other approaches like proton or carbon ion therapy with their superior dose distributions [15, 16] are a promising treatment modality for soft tissue sarcoma in the preoperative setting, potentially being able to reduce wound complications. In addition, aiming for a minimum dose in the prospective surgery incision area of the skin could be realized very well with protons and carbon ions and thus, potentially reduce wound complications.

Heavy ions are considered as a good treatment option for tumors of low radiosensitivity as sarcomas. This being exemplified in a prospective phase I/II trial of 17 unresected bone and soft tissue sarcomas of the extremities with high 5 year-LC-rates of 76% [25]. So far, data on proton or carbon ion therapy in the treatment of soft tissue sarcoma is relatively limited. A prospective trial with proton re-irradiation on 23 patients with recurrent and secondary soft tissue sarcoma in different locations (10 patients with extremity soft tissue sarcoma) showed that re-irradiation with protons is being well tolerated and safe in this treatment setting with wound complications in up to 15% of cases [26]. In a recent ASCO abstract, a retrospective study of 18 patients with extremity soft tissue sarcoma treated with preoperative pencil beam proton radiotherapy, 39% developed wound complications (intraoperative debridement (25%) being the most common one), which is comparable to data from prospective studies on photon irradiation [27]. These data suggest that particle radiotherapy has at least comparable toxicities to photon irradiation and is assumed to be safe in the use of sarcomas of the extremities.

With the advances in radiotherapy treatment techniques, hypofractionation is growing in popularity for the radiotherapy of sarcoma [28, 29]. The exact α/β ratio of extremity soft tissue sarcomas is not established yet but nonetheless a high fractionation sensitivity is currently assumed (α/β < 4–5), and thus, hypofractionation seems to be beneficial in regard to the radiobiological response of sarcoma cells [30]. The technical advantages of particles are utilized in order to compensate for the potential aggravation of side effects by the implementation of hypofractionation in this protocol. Potential risk lies in the uncertainties in the definition of the equivalent total dose to the standard normofractionated schedule of 50 Gy in 25 fractions. Additionally, in this trial, the overall radiotherapy time is reduced by half through hypofractionation. This might not only improve the patient’s quality of life but also reduce the risk of progression in the timeframe prior to surgery.

### Trial status

Protocol Version 1.2, June 23, 2020, the recruitment began on 21.06.2021, the approximate date of recruitment completion is July, 2023.

## Data Availability

No unpublished datasets were used and/or analyzed for the study protocol. Any data is available from the corresponding author on reasonable request.

## Abbreviations

CTCAE: Common Terminology Criteria for Adverse Events
CTV: Clinical target volume
DFS: Disease free survival
DSMB: Data and Safety Monitoring Board
GTV: Gross tumor volume
HIT: Heidelberg Ion-Beam Therapy Center
ICA-r: Independent component analysis with reference
IMRT: Intensity modulated radiotherapy
ITT: Intention to treat
LC: Local control
LPFS: Local progression free survival
OS: Overall survival
PI: Principal Investigator
PFS: Progression free survival
PTV: Planning target volume
QoL: QUALITY of life
RBE: Relative biological effectiveness
RT: Radiotherapy
